# Naturally Occurring Bicoumarin Compound Daphnoretin Inhibits Growth and Induces Megakaryocytic Differentiation in Human Chronic Myeloid Leukemia Cells

**DOI:** 10.3390/cells11203252

**Published:** 2022-10-16

**Authors:** Yu-Chuen Huang, Chun-Ping Huang, Chin-Ping Lin, Kai-Chien Yang, Yu-Jie Lei, Hao-Pei Wang, Yueh-Hsiung Kuo, Yu-Jen Chen

**Affiliations:** 1 School of Chinese Medicine, China Medical University, Taichung 40402, Taiwan; 2 Department of Medical Research, China Medical University Hospital, Taichung 40447, Taiwan; 3 Integration Center of Traditional Chinese and Modern Medicine, Department of Chinese Medicine, Hualien Tzu Chi Hospital, Hualien 97002, Taiwan; 4 Department of Medical Research, MacKay Memorial Hospital, New Taipei City 25160, Taiwan; 5 Department of Public Health, China Medical University, Taichung 40402, Taiwan; 6 Department of Chinese Pharmaceutical Sciences and Chinese Medicine Resources, China Medical University, Taichung 40402, Taiwan; 7 Department of Biotechnology, Asia University, Taichung 41354, Taiwan; 8 Department of Radiation Oncology, MacKay Memorial Hospital, Taipei 10449, Taiwan; 9 Department of Artificial Intelligence and Medical Application, MacKay Junior College of Medicine, Nursing and Management, Taipei 11260, Taiwan

**Keywords:** daphnoretin, differentiation, megakaryocyte, protein kinase C, myeloid leukemia

## Abstract

Daphnoretin extracted from the stem and roots of *Wikstroemia indica* (L.) C.A. Mey has been shown to possess antiviral and antitumor activities. Herein, we hypothesized that daphnoretin might induce megakaryocytic differentiation, thereby inhibiting the proliferation of cells and serving as a differentiation therapy agent for chronic myeloid leukemia (CML). Daphnoretin-treated K562 and HEL cells were examined for growth inhibition, cell morphology, and megakaryocyte-specific markers. Potential mechanisms of megakaryocytic differentiation of daphnoretin-treated K562 cells were evaluated. The results showed that daphnoretin inhibited the growth of K562 and HEL cells in a dose- and time-dependent manner. Flow cytometry analyses revealed that daphnoretin treatment slightly increased the proportion of sub-G1 and polyploid cells compared to that of dimethyl sulfoxide (DMSO)-treated control cells. Morphological examination showed that daphnoretin-treated K562 and HEL cells exhibited enlarged contours and multinucleation as megakaryocytic characteristics compared to DMSO-treated control cells. Daphnoretin treatment also dramatically enhanced the expression of megakaryocytic markers CD61 and CD41. Under optimal megakaryocytic differentiation conditions, daphnoretin increased the phosphorylation of STAT3 but not STAT5. In summary, daphnoretin inhibited cell growth and induced megakaryocytic differentiation in K562 and HEL cells. The efficacy of daphnoretin in vivo and in patients with CML may need further investigations for validation.

## 1. Introduction

Chronic myeloid leukemia (CML) is a myeloproliferative neoplasm characterized by the Philadelphia chromosome with a translocation between chromosomes 9 and 22. This chromosomal translocation generates an oncogenic Bcr-Abl fusion protein, which constitutively produces an active tyrosine kinase [1]. Tyrosine kinase inhibitors have been approved for first-line or second-line therapy for CML; however, there are limitations of their use. Treatment with second-generation tyrosine kinase inhibitors in the accelerated or blast phase provides a poor prognosis, and alternative therapies should be considered for patients without a complete hematological response [2,3]. In differentiation therapy, the differentiation of blast cells into mature cells is induced, thereby inhibiting the proliferation of cancer cells. Retinoic acid and arsenic trioxide are successful clinically used agents for induction therapy to treat acute promyelocytic leukemia [4,5]. However, an efficient differentiation therapy strategy for CML is still lacking. Nobiletin, a polymethoxyflavone phytochemical, exerts anti-leukemic effects and promotes megakaryocytic differentiation; when combined with imatinib, it exerts a synergistic cytotoxic effect on cancer cells [6]. In addition, atypical protein kinase C has also been reported to be a leukemic suppressor and is a new target for anti-cancer treatments [7]. 

K562 and human erythroleukemia (HEL) cells derived from patients with CML and erythroleukemia, respectively, have pluripotency in terms of both erythroid and megakaryocytic differentiation [8,9,10] and have been widely used to investigate the regulation of differentiation and differentiation inducers in vitro. Treatment of K562 and HEL cells with phorbol esters, such as 12-O-tetradecanoyl-phorbol-13-acetate (TPA), leads to megakaryocytic characteristics and the loss of erythroid properties [11,12], resulting in irreversible cell growth arrest, increased cell size and DNA content, and increased cell-cell and cell-substrate adhesion. The hallmarks of megakaryocytic differentiation are endoreduplication accompanied by the expression of cell surface specific markers, such as CD41 (GPIIa or αIIb) and CD61 (GPIIIa or β3) [13,14]. Phorbol esters have the potential to be developed as agents for differentiation therapy for CML patients; however, there are safety concerns regarding their tumor-promoting activities. Thrombopoietin, also known as c-Mpl ligand, is the most potent cytokine for stimulating the proliferation and differentiation of megakaryocyte progenitor cells; therefore, it is a major regulator of megakaryopoiesis and platelet formation [15,16]. However, thrombopoietin does not provide promising outcomes in clinical use, as reported in in vitro studies. Therefore, developing novel inducers of megakaryocytic differentiation is still an urgent task.

*Wikstroemia indica* (L.) C. A. Mey., a member of the Thymelaeaceae family, has long been widely used as a traditional Chinese medicine and folk remedy plant for the treatment of syphilis, arthritis, bronchitis, and many type of cancers [17,18]. Daphnoretin (7-hydroxyl-6-methoxy-3,7-dicoumarylether) is a naturally occurring bicoumarin compound abundantly present in the stem and roots of *W. indica* C.A. Mey. with multiple functions [19]. Previous reports have indicated that daphnoretin is a protein kinase C (PKC) activator that possesses antiviral activity by functioning on the PKC pathway to suppress hepatitis B virus expression in human hepatoma cells [20,21]. Moreover, daphnoretin can regulate the immune response by modulating dendritic cell development toward atypical maturation with impaired allostimulatory functions [22]. In addition, it has exhibited anticancer activity against several tumors, including Ehrlich ascites tumor cells [23], leukemia cells [19,24], cervical cancer HeLa cells [25], lung adenocarcinoma A549 cells [26], and colon cancer HCT116 cells [27]. Although these studies have shown the potential of daphnoretin in treating specific cancers, there is no evidence of its inhibitory or differentiation-inducing effect on human myeloid leukemia cells. Here, we studied the inhibitory effect of daphnoretin and the effect of dephnoretin-induced megakaryocytic differentiation using human K562 and HEL cells.

## 2. Materials and Methods

### 2.1. Reagents

The 12. -O-Tetradecanoylphorbol-13-acetate (TPA), midostaurin, and dimethylsulfoxide (DMSO) were purchased from Sigma-Aldrich (St. Louis, MO, USA). Trypan blue solution was purchased from ThermoFisher (0.4%, Thermo Fisher Scientific, Runcorn, Cheshire, UK). Liu’s stain A and B solution was purchased from Tonyar Biotech. Inc. (Taoyuan City, Taiwan). The Cell Counting Kit-8 (CCK-8) was purchased from MCE USA (MedChemExpress, Princeton, NJ, USA).

### 2.2. Preparation of Daphnoretin

Daphnoretin was synthesized and provided for the experiments by Prof. Yueh-Hsiung Kuo from the China Medical University, Taiwan, as described previously [21]. Briefly, daphnoretin was extracted from the ethanolic and n-hexane extracts of the stem and roots of W. indica (L.) C.A. Mey. and isolated using silica gel column chromatography. According to the chemical and spectroscopic properties, it was identified as daphnoretin. Before use, daphnoretin was dissolved in DMSO.

### 2.3. Cell lines and Culture

The K562 and HEL 92.1.7 cells were obtained from the Bioresource Collection and Research Center of the Food Industry Research and Development Institute (Hsinchu, Taiwan). Cells were cultured in RPMI 1640 (Gibco BRL, Rockville, MD, USA) medium and supplemented with 10% of fetal bovine serum (Cell Biologics, Inc., Chicago, IL, USA), 2 mM of L-glutamine, 100 IU/mL of penicillin, and 100 mg/mL of streptomycin. Cultures were maintained at 37 °C in a humidified 5% CO_2_ incubator.

### 2.4. Cell Growth and Cell Viability Measurement

K562 and HEL cells at a density of 1 × 10^5^ cells/mL were treated with DMSO, 0.25 or 1.00 μM daphnoretin, or 0.10 μM TPA for 24, 48, and 72 h. Trypan blue dye exclusion assays were used to measure the number of viable cells. The CCK-8 assay was used to measure cell viability. K562 and HEL cells were cultured in 96-well plates (1×10^4^ cells/well) and were then treated with DMSO, 0.25 or 1.00 μM daphnoretin, or 0.10 μM TPA for 24, 48, and 72 h. This was followed by incubation with the CCK-8 solution for 3 h at 37 °C. Cell viability was measured as the absorbance at 450 nm using a microplate reader. 

### 2.5. Morphological Examination

The cells were cytocentrifuged (Cytospin 4, Shandon Southern Instrument Inc., Sewelicky, PA, USA) onto microscope slides and then subjected to staining with Liu’s stain, and the cell morphology was viewed using a light microscope (Olympus BX51, Tokyo, Japan). Two hundred cells in randomly chosen fields were counted and examined under microscopy, and the proportion of multinucleated cells was expressed as a percentage. 

### 2.6. Surface Marker Expression Assessed by Immunofluorescent Analysis

Immunofluorescent analysis was used to detect megakaryocyte-specific cell surface markers after treating cells with DMSO or 1.0 μM daphnoretin for 72 h, as described previously [28]. In brief, cells were stained with fluorescein isothiocyanate (FITC)-conjugated CD41, phycoerythrin (PE)-conjugated CD61 (Elabscience, Houston, TX, USA), and FITC-conjugated CD42a (Thermo Fisher Scientific, Waltham, MA, USA) antibodies. Cells were then incubated in Hoechst 33,342 (Thermo Fisher Scientific, Waltham, MA, USA) to visualize cell nuclei. Fluorescence intensities were evaluated using an Axio imager A2 microscope with an Axiocam 506 color camera system (Carl Zeiss, Oberkochen, Germany).

### 2.7. Surface Marker Expression Assessed by Flow Cytometry Analysis

Flow cytometry was used to detect the specific megakaryocyte antigens on the cell surface after the induction of differentiation, as described previously [28]. In brief, cells were stained with fluorescein Alexa Flour 647 (AF647)-conjugated CD41, phycoerythrin (PE)-conjugated CD61 (Elabscience, Houston, TX, USA), and isothiocyanate (FITC)-conjugated CD42a (Thermo Fisher Scientific, Waltham, MA, USA) antibodies, followed by analysis using a Novocyte 3000 flow cytometer and NovoExpress software (ACEA Bioscience Inc., San Diego, CA, USA).

### 2.8. Cell Cycle Analysis

Cell cycle distribution was analyzed on a FACSCalibur flow cytometer using ModFit 3.0 software (BD Biosciences, San Jose, CA, USA), as previously described [28,29]. In brief, cells fixed with 70% ethanol for at least 30 min at 4 °C were washed with PBS and then stained with 10 mg/mL propidium iodide in the dark for 30 min at 37 °C.

### 2.9. RNA Preparation and Quantitative Real-Time Polymerase Chain Reaction (qRT-PCR)

Total RNA was isolated from treated cells using the PureLink RNA mini kit (Thermo Fisher Scientific, Waltham, MA, USA). RNA concentration was measured using a NanoDrop ND-1000 spectrophotometer (Thermo Fisher Scientific, Waltham, MA, USA). In total, 0.8 μg of total RNA was reverse transcribed using the High-Capacity cDNA Reverse Transcription kit (Thermo Fisher Scientific, Waltham, MA, USA), as previously described [30]. The mRNA levels were quantified using SYBR Green Master Mixes on the LightCycler 480 System (Roche, Mannheim, Germany), as described in a previous study [31]. The mRNA expression of specific megakaryocyte markers and PKC isozymes was normalized to GAPDH expression, calculated by the ΔCt method. The primer pairs for qRT-PCR are shown in Table 1.

### 2.10. Western Blotting

Proteins were extracted from K562 cells cultured with DMSO, 1.0 μM daphnoretin, 0.5 μM midostaurin (a PKC inhibitor), or TPA at 0.1 μM for 24, 48, and 72 h using radioimmunoprecipitation assay (RIPA) lysis buffer (Sigma Aldrich, St. Louis, MI, USA) with protease and phosphatase inhibitors (Roche, Indianapolis, IN, USA). Western blotting was performed according to a previous study [32]. Membranes were incubated overnight at 4 °C with the following primary antibodies at a 1:1000 dilution: anti-JAK2, anti-phospho-JAK2 (Tyr1007/1008), anti-STAT5, anti-phospho-STAT5 (Tyr694), anti-phospho-STAT3 (Tyr705) antibody (all from Cell Signaling Technology, MA, USA), and anti-STAT3 antibody (BD Biosciences, San Jose, CA, USA). Protein expression was detected using the ChemiGenius Bio Imaging System (Syngene, Frederick, MD, USA). The intensity of the bands was quantified using ImageJ software and levels were normalized to those of the internal control, β-actin.

### 2.11. Statistical Analysis

Statistical analyses were performed using IBM SPSS version 22 (IBM Co., Armonk, NY, USA). Results are presented as the mean ± standard deviation (SD), and mean differences were compared using a one-way analysis of variance (ANOVA) followed by Dunnett’s post-hoc test. *p*-values less than 0.05 were considered statistically significant.

## 3. Results

### 3.1. Daphnoretin Inhibited the Cell Growth

**Figure 1:**
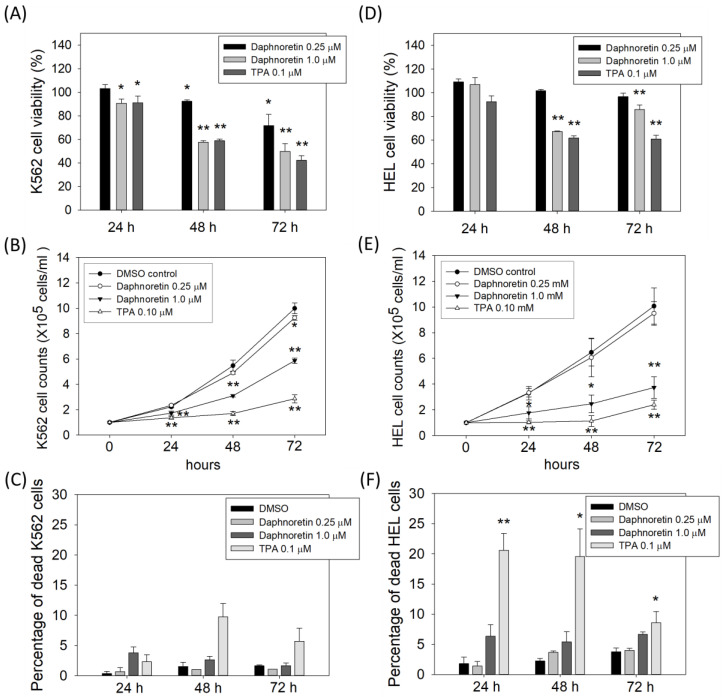
Effect of daphnoretin on the growth of K562 and HEL cells. Cells were treated with DMSO or 0.25, 1 µM daphnoretin, 0.1 µM TPA after 24–72 h. (**A**,**D**) Cell viability was determined using CCK-8 assay. The data represent the mean ± SD of three independent experiments, and the ratio of cell viability was presented as 100% in the DMSO group. * *p* < 0.05, ** *p* < 0.01 vs. DMSO-treated sample (ANOVA test followed by Dunnett’s post-hoc test). (**B**,**E**) The number of viable cells was counted using trypan blue exclusion assay. The data represents the mean ± SD of three independent experiments * *p* < 0.05, ** *p* < 0.01 vs. DMSO-treated sample (ANOVA test followed by Dunnett’s post-hoc test). (**C**,**F**) The percentage of dead cells was determined using trypan blue exclusion assay. The data represents the mean ± SD of three independent experiments * *p* < 0.05, ** *p* < 0.01 vs. DMSO-treated sample (ANOVA test followed by Dunnett’s post-hoc test).

To evaluate the anticancer activity of daphnoretin, K562 and HEL cells were treated with DMSO (vehicle control), daphnoretin, or TPA (as a positive control) for 24–72 h. Results from the CCK-8 assay showed that daphnoretin decreased cell viability in a dose-dependent manner in K562 (Figure 1A) and HEL (Figure 1D) cells. In addition, the inhibitory effect of daphnoretin on cell proliferation was further determined by trypan blue exclusion assay. Results showed that the number of viable daphnoretin-treated K562 and HEL cells was significantly reduced in a dose- and time-dependent manner compared with that of DMSO-treated cells, as shown in Figure 1B,E. The low percentage of dead cells in 0.25 μM daphnoretin-treated K562 (0.7%, 1.0%, and 1.1% for 24–72 h) and HEL (1.4%, 3.7%, and 4.0% for 24–72 h) cells is shown in Figure 1C,F. These results indicate that daphnoretin treatment inhibited the cell growth of K562 and HEL cells. 

### 3.2. Effect of Daphnoretin on the Cell Cycle Distribution of K562 and HEL Cells

**Figure 2:**
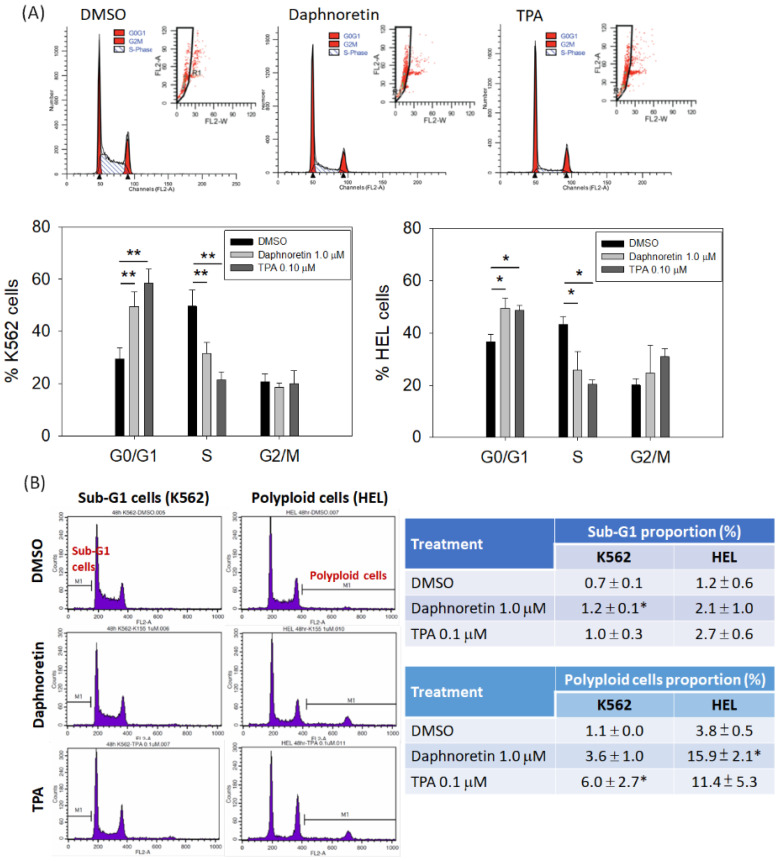
Effects of daphnoretin on the cell cycle distribution. Cells were treated with DMSO, daphnoretin (1.0 μM), or 12-O-tetradecanoyl-phorbol-13-acetate (TPA; 0.1 µM), followed by flow cytometric analysis. (**A**) Representative gating strategy of flow cytometry analysis in cell cycle distribution; (**B**) proportion of sub-G1 and polyploid cells at 48 h; * *p* < 0.05, ** *p* < 0.01 vs. DMSO-treated sample (ANOVA test followed by Dunnett’s post-hoc test).

Next, we evaluated the cell cycle distribution of K562 and HEL cells treated with daphnoretin (1 µM) or TPA (0.1 µM) for 24–48 h using flow cytometry. As shown in Figure 2A, the proportion of G0/G1 phase cells was significantly increased and was accompanied by a decrease in the proportion of S-phase cells in the daphnoretin and TPA-treated groups, as compared with results in DMSO-treated cells, for 24 h. In addition, the proportion of dead cells (sub-G1 phase) was slightly increased among daphnoretin-treated K562 and HEL cells, compared with that in DMSO-treated cells, at 48 h (daphnoretin vs. DMSO-treated K562 cells: 1.2 ± 0.1% vs. 0.7 ± 0.1%, *p* < 0.05; daphnoretin vs. DMSO-treated HEL cells: 2.1 ± 1.0% vs. 1.2 ± 0.6%), as shown in Figure 2B. Moreover, daphnoretin induced polyploidy in K562 (3.6 ± 1.0%) and HEL (15.9 ± 2.1%, *p* < 0.05) cells compared with that with DMSO treatment (Figure 2B). These results indicate that daphnoretin treatment may increase the proportion of dead and polyploid cells in K562 and HEL cells by a small amount.

### 3.3. Daphnoretin-Induced Morphological Changes and Megakaryocyte-Specific Marker Expression

**Figure 3:**
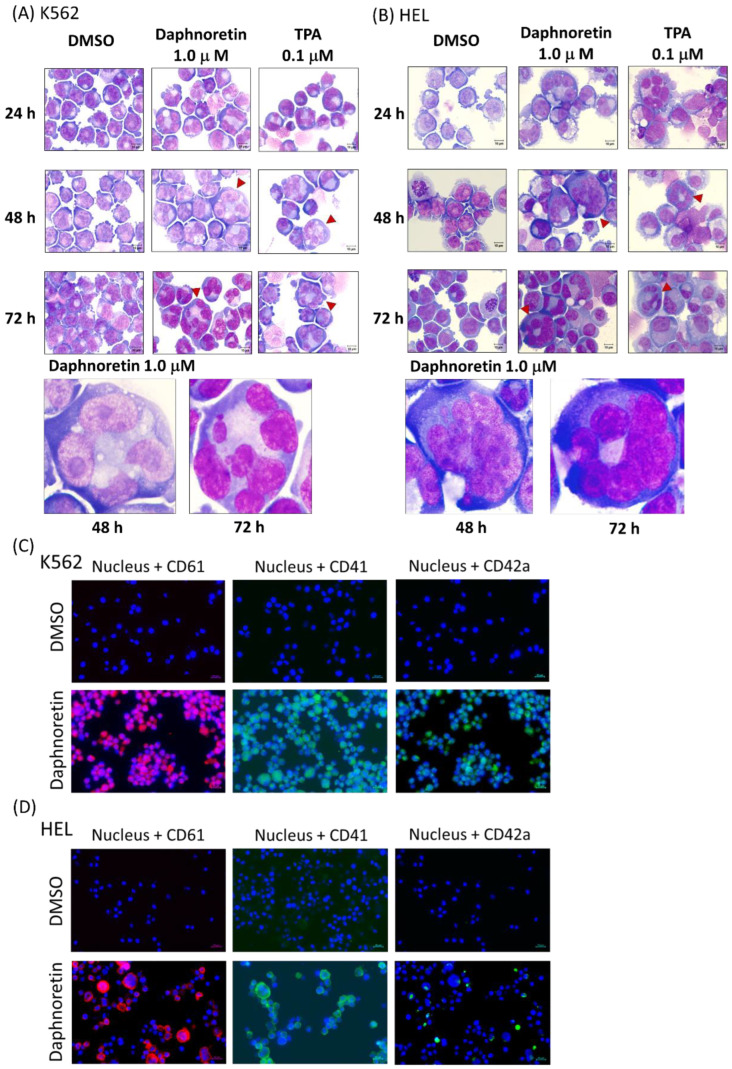
Morphological examination of (**A**) K562 and (**B**) HEL cells treated with 1 μM daphnoretin or 0.1 µM TPA for 24–72 h. Cells were stained with Liu’s stain for morphological observation at the original magnification of 1000×. Scale bars represent 10 μM, and red arrowhead indicates enlargement of cells with multiple nuclei. Immunofluorescent analysis of (**C**) K562 and (**D**) HEL cells treated with DMSO or 1 μM daphnoretin for 72 h. Cells were expressed CD61 (red), CD41 (green), and CD42a (green). The nuclei were stained with Hoechst 33,342 (blue). Scale bars represent 20 μM.

Cells were treated with daphnoretin (1 µM) or TPA (0.1 µM) for 24–72 h and then observed under a light microscope. The results showed daphnoretin-induced cell size increased and the appearance of multiple nuclei in K562 (Figure 3A) and HEL cells (Figure 3B). The percentage of multinucleated cells was significantly increased in daphnoretin-treated and TPA-treated K562 and HEL cells compared with that in DMSO-treated cells for 24–72 h, as shown in Table 2. In addition, immunofluorescence analyses showed that megakaryocyte-specific markers CD61, CD41, and CD42a were more strongly detected in daphnoretin-treated K562 and HEL cells compared to DMSO-treated control cells after 72 h (Figure 3C,D).

### 3.4. Daphnoretin Increases the Expression of Megakaryocyte-Specific Markers

**Figure 4:**
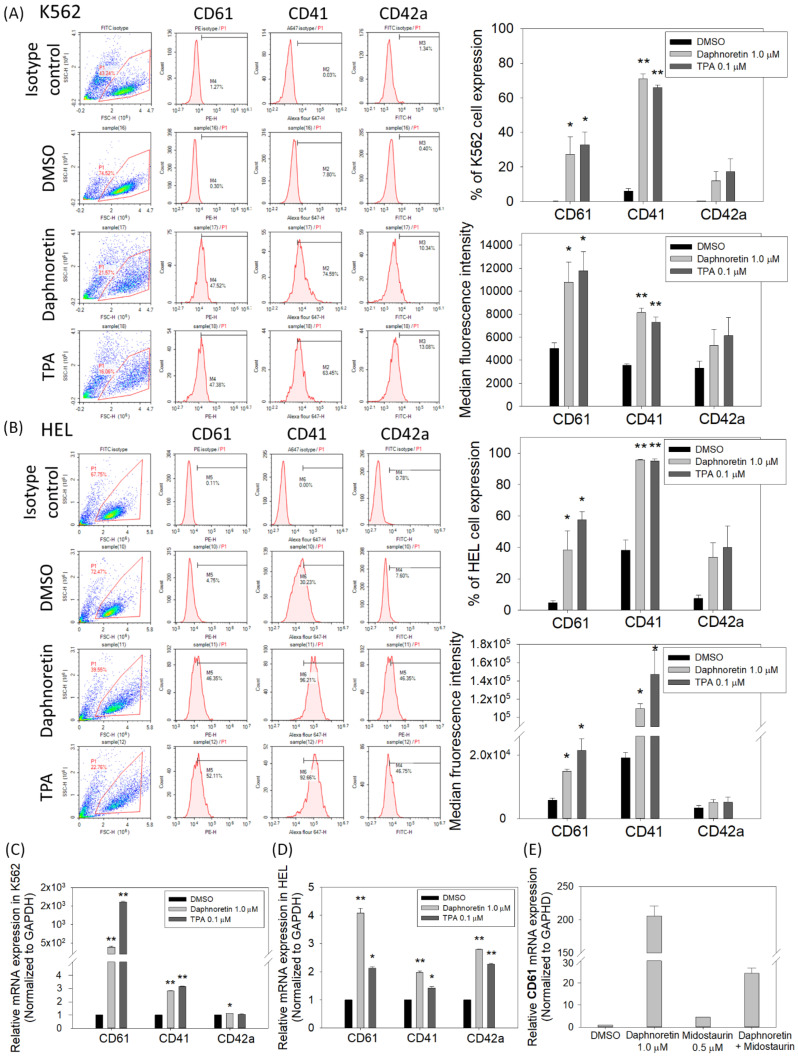
Expression of CD61, CD41, and CD42a megakaryocyte-specific markers analyzed using flow cytometry in (**A**) K562 and (**B**) HEL cells. The data represent the percentages of positive cells and mean fluorescence intensity from three independent experiments. Relative messenger RNA (mRNA) expression level of megakaryocyte-specific markers in (**C**) K562 and (**D**) HEL cells. (**E**) The mRNA expression level of CD61 in K562 cells treated with DMSO, daphnoretin (0.1 μM), midostaurin (0.5 μM), or pretreated with midostaurin (0.5 μM) for 30 min followed by treatment with daphnoretin (0.1 μM) for 24 h. The mRNA expression levels of CD61, CD41, and CD42a were measured using qRT-PCR assay and normalized to the GAPDH control expression. The data represent the mean ± SD of three independent experiments. * *p* < 0.05, ** *p* < 0.01 vs. DMSO-treated sample (ANOVA followed by Dunnett’s post-hoc test).

To analyze the effect of dephnoretin on the megakaryocyte-specific markers expression, cells were treated with DMSO, daphnoretin (1 µM), or TPA (0.1 µM) for 72 h. The treated cells were harvested and stained with CD61, CD41, or CD42a, followed by flow cytometric analysis. As shown in Figure 4A, the percentage of CD61 and CD41-positive cells and median fluorescence intensity (MFI) significantly increased in the daphnoretin and TPA-treated K562 cells compared with that in the DMSO control (CD61: *p* < 0.05, CD41: *p* < 0.001). The same phenomenon was observed in daphnoretin-and TPA-treated HEL cells for the percentage of CD61 and CD41-positive cells and MFI was significantly increased compared to the DMSO control (Figure 4B, *p* < 0.05). In addition, qPCR was also used to assess the megakaryocytic differentiation markers expression. As shown in Figure 4C,D, daphnoretin-treated K562 and HEL cells significantly increased CD61, CD41, and CD42a mRNA expression for 72 h (*p* < 0.05). CD42a mRNA expression was dramatically increased in daphnoretin-and TPA-treated HEL cells. We further treated K562 cells with a combination of daphnoretin and the PKC inhibitor midostaurin, and the daphnoretin induced-CD61 expression was reversed by midostaurin, as shown in Figure 4E.

### 3.5. JAK2 and STAT3/STAT5 Expression in Daphnoretin-Treated Cells

**Figure 5:**
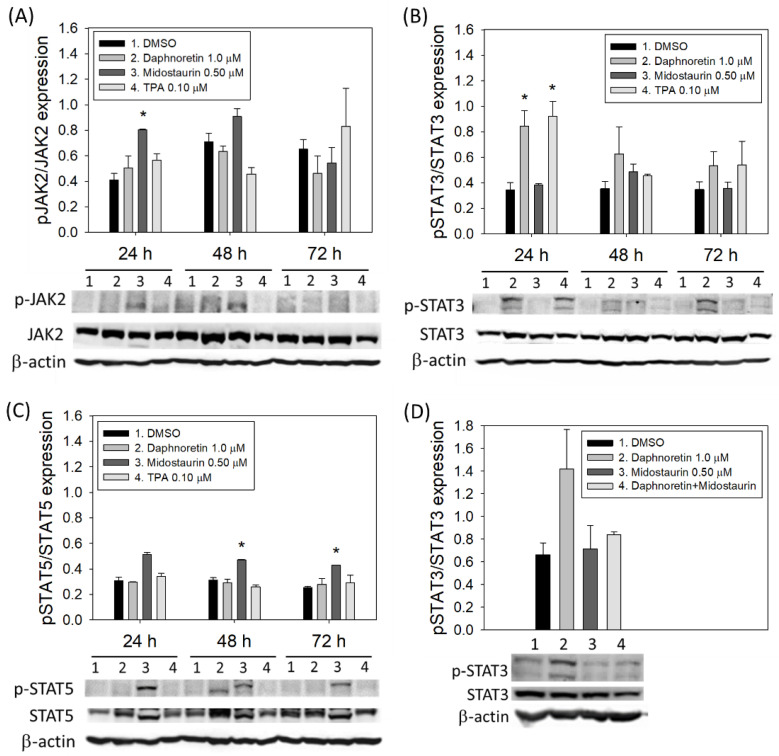
Immunoblot analysis of K562 cells treated with DMSO, daphnoretin (1 µM), midostaurin (0.5 μM), or 12-O-tetradecanoyl-phorbol-13-acetate (TPA; 0.1 µM) for 24–72 h. Normalization of (**A**) JAK2, (**B**) STAT3, (**C**) STAT5, as well as (**D**) expression levels of STAT3 after combined daphnoretin and midostaurin, relative to levels of the internal control, β-actin. Data are presented as the mean ± SD of three independent experiments. * *p* < 0.05 vs. DMSO-treated sample (ANOVA test followed by Dunnett’s post-hoc test).

Activation of JAK2 and STAT3/STAT5 is considered one of the important signaling pathways involved in the differentiation of megakaryocytes. We therefore evaluated the expression of JAK2 and STAT3/5 in K562 cells treated with 1 µM daphnoretin or 0.5 μM midostaurin or 0.1 µM TPA for 24–72 h. As shown in Figure 5, daphnoretin treatment increased the phosphorylation of STAT3 (p-STAT3) expression, but not p-JAK2 and p-STAT5. The expression of p-STAT5 was increased after midostaurin treatment (Figure 5C). When K562 cells were treated with a combination of daphnoretin and midostaurin, the daphnoretin-increased p-STAT3 expression was attenuated by midostaurin, as shown in Figure 5D. 

### 3.6. Relative mRNA Expression of the Different PKC Isozymes under Different Treatment

**Figure 6:**
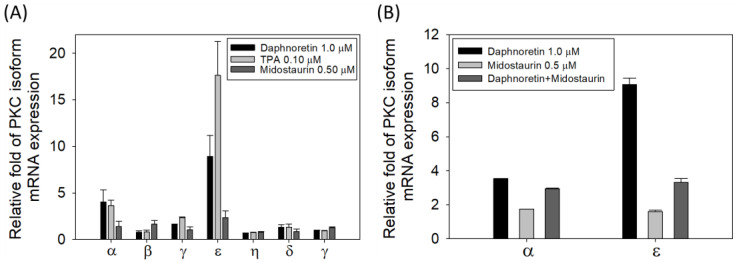
Relative mRNA expression of (**A**) different PKC isozymes with different treatments, (**B**) PKCα/PKCε with daphnoretin and midostaurin combined treatment. The mRNA expression was quantified by reverse transcription and qPCR using the Δct method. The mRNA levels of PKC were measured and normalized to the levels of the GAPDH control. Data represent the means ± SDs of three independent measurements.

Since daphnoretin is reported as a PKC activator, we examined the mRNA expression patterns of the PKC isozymes in K562 cells treated with DMSO, daphnoretin (1 µM), TPA (0.1 µM), or midostaurin (0.5 μM) for 24 h. As shown in Figure 6A, the most abundant PKC isozyme in daphnoretin and TPA-treated cells was PKCε, followed by PKCα, and the expression of PKCε and PKCα was attenuated by daphnoretin and midostaurin combined treatment (Figure 6B). However, no obvious abundant PKC isozyme mRNA expression level was observed in midostaurin-treated cells. This result indicates that daphnoretin might increase PKCε and PKCα expression.

## 4. Discussion

Here, we demonstrated that daphnoretin can inhibit growth and induce megakaryocytic differentiation in human K562 and HEL cells. A previous in vivo study indicated that daphnoretin can inhibit the growth of P-388 lymphocytic leukemia in mice [19]. In addition, less than 10% cell death was reported after 48 h of daphnoretin treatment of K562 cells at a concentration of 0.07 mg/mL (approximately 0.2 μM) [24]. The results indicate that daphnoretin has antitumor activity and could have a novel therapeutic effect against myeloid leukemia or lymphocytic leukemia. Thus, further investigations are required to characterize and optimize its clinical applications.

Daphnoretin treatment leads to the expression of megakaryocytic characteristics. Distinct cellular features were observed after K562 and HEL treatment with daphnoretin for 48–72 h, including increases in cell size and multinucleated cells. In addition, megakaryocyte differentiation is characterized by the increased expression of CD41 and CD61. Surface CD41 and CD61 expression significantly increased after daphnoretin treatment through quantitation of surface protein using flow cytometric analysis and mRNA using qPCR. Previous reports indicated that the activity of PKC can act as a developmental switch to control erythroid and megakaryocytic differentiation [33]. Since daphnoretin is reported to be a PKC activator [21], it is not surprising that it could induce megakaryocytic differentiation. The mRNA expression profiles revealed that PKCε and PKCα were the most expressed isozymes in daphnoretin-treated cells. However, the exact subcellular location of PKC activation and subsequent activation of downstream proteins in daphnoretin-treated K562 cells needs further study. In terms of the megakaryocytic differentiation signaling pathway induced by daphnoretin, we observed that the phosphorylation of STAT3, but not STAT5, was expressed after daphnoretin treatment. This is consistent with a previous report indicating that STAT3 is required for the effective expansion of megakaryocytic progenitor cells in the early stages of megakaryopoiesis [34]. In addition, we observed that combination of daphnoretin and the PKC inhibitor midostaurin partially reversed the expression of CD61, pSTAT3, and PKCε/PKCα, suggesting that CD61 and pSTAT3 may play a role in dephnoretin-mediated megakaryocytic differentiation. However, midostaurin did not reduce the daphnoretin-induced formation of multi-nucleated cells. Instead, daphnoretin increased both the enlargement of cell size and the extensive formation of multi-nucleation. It might implicate the role of daphnoretin as a PKC activator to partially reverse the formation of multi-nucleation induced by the PKC inhibitor midostaurin (data not shown). As midostaurin is a multi-targeted protein kinase inhibitor, a specific PKC inhibitor (e.g., bisindolylmaleimide) must be used to examine whether PKC is involved in daphnoretin-induced cell differentiation. In addition, we did not observe a significant change in JAK2 expression in K562 cells after daphnoretin treatment. The phosphorylation of JAK2 can be induced by TPO, resulting in the phosphorylation of downstream targets, including activation of the transcription factors STAT3/STAT5 [35]. Signaling through these pathways causes the downstream activation of megakaryocyte-specific transcription factors and regulation of the expression of megakaryocyte-specific genes. Since the TPO receptor c-Mpl is not expressed in K562 cells [36], daphnoretin probably activates a novel pathway for the induction of megakaryocytic differentiation. However, further in vivo experiments are needed to investigate whether daphnoretin can induce platelet formation from differentiated megakaryocytes. 

Although K562 and HEL cells are an ideal cellular model for studying megakaryocytic differentiation and globin gene expression [37], this study was limited by only focusing on a cellular model to examine the effect of daphnoretin on the K562 and HEL cells. Further studies are required to validate the effect of daphnoretin using other primary cultures of CML cells and animal models. The long-term primary culture of leukemia cells from the bone marrow of patients remains a challenge to overcome. The BALB/c mouse CML model requires the processing of retrovirus transduction and bone marrow transplantation [38], which is relatively highly manipulated and costly in terms of the time and resources required. However, the effects of daphnoretin on CML in animal and human studies still warrant further investigation.

## 5. Conclusions

In conclusion, daphnoretin inhibited human K562 and HEL cell growth and induced megakaryocytic differentiation. The efficacy of daphnoretin in vivo and in patients with CML may need further investigations for validation.

## Figures and Tables

**Table 1:** The primer pairs used for quantitative real-time PCR.

Genes	Primers
CD41	5′ -TGCTGCTCACCATCCTGGTC 5′ -AACCCAAAGCTTGGAGGCAAC
CD42a	5′ -TCTGTATCAGAAGCCCTGTCTTCAC 5′ -GCATCGGGAGCTTTGTCTTG
CD61	5′ -TGTATGGGACTCAAGATTGGA 5‘ -AGGGATGGCTATTAGGTTCA
PKC α	5′ -CAAGGTTCATGCAGCCCAAC 5′ -ACTGTGTCCCTGGCAAAACA
PKC β	5′ -GACCAAACACCCAGGCAAAC 5′ -GATGGCGGGTGAAAAATCGG
PKC γ	5′ -GAGATCCCGCCTCCTTTCAG 5′ -CTGGGGTGCAGGATATGACG
PKC ε	5′ -CGGCGAGGAAATACATGCAC 5′ -GGGCAGGAATGAAGAACCGA
PKC η	5′ -GGTGCTGAAGAAGGACGTGA 5′ -AAAACAGACGATCGGGGGTC
PKC δ	5′ -TGGTTGGTGCGTTGTAGCAG 5′ -TAGGAGTTGAAGGCGATGCG
PKC θ	5′ -AAACCTCAAGGCCGAATGC 5′ -AGAAGGTGGCAGTGAACTCG
GAPDH	5′ -ATGAGAAGTATGACAACAGCCT 5′ -AGTCCTTCCACGATACCAAAGT

**Table 2:** Proportion of multiple nuclei cells in daphnoretin and TPA-treated K562 and HEL cells.

		Multiple Nuclei Cell (%)		
		DMSO	Daphnoretin (1.0 μM)	TPA (0.1 μM)
24 h	K562	1.5 ± 1.5	44.5 ± 3.3 *	51.4 ± 4.8 *
	HEL	3.8 ± 0.3	25.8 ± 5.3 *	22.5 ± 6.7 *
48 h	K562	7.0 ± 1.5	44.7 ± 10.9 *	54.4 ± 13.8 *
	HEL	3.1 ± 1.4	34.1 ± 4.3 **	39.4 ± 4.2 **
72 h	K562	6.2 ± 0.3	43.3 ± 2.1 *	67.4 ± 4.4 *
	HEL	6.0 ± 1.2	34.8 ± 5.0 **	39.6 ± 5.8 **

* *p* < 0.01, ** *p* < 0.001 compared with DMSO-treated cells.
